# Near-infrared bioluminescence imaging of two cell populations in living mice

**DOI:** 10.1016/j.xpro.2021.100662

**Published:** 2021-07-07

**Authors:** Giorgia Zambito, Laura Mezzanotte

**Affiliations:** 1 Erasmus Medical Center, Dept. of Radiology and Nuclear Medicine, Rotterdam 3015 CE, the Netherlands; 2 Erasmus Medical Center, Dept. of Molecular Genetics, Rotterdam 3015 CE, the Netherlands

**Keywords:** Model Organisms, Molecular/Chemical Probes, Biotechnology and bioengineering

## Abstract

Multicolor bioluminescence imaging using near-infrared emitting luciferases is an attractive application to detect two cell populations within one animal model. Herein, we describe how to distinguish dual-color bioluminescent signals co-localized in the same compartment. We tested CBG2 click beetle (λ = 660 nm) and CBR2 click beetle (λ = 730 nm) luciferases paired with NH_2_-NpLH2 luciferin. Following a spectral unmixing algorithm, single spectral contributions can be resolved and quantified, enabling the visualization of multiple cell types in deep tissue by injection of a single substrate.

For complete details on the use and execution of this protocol, please refer to Zambito et al. (2020).

## Highlights

• A protocol for dual-color bioluminescence imaging in deep tissue is provided
• A step-by-step spectral unmixing tool is applied for spectral properties of luciferases
• Co-localized green and red signals can be separated and quantified *in vivo*

## Graphical abstract

**Figure:**
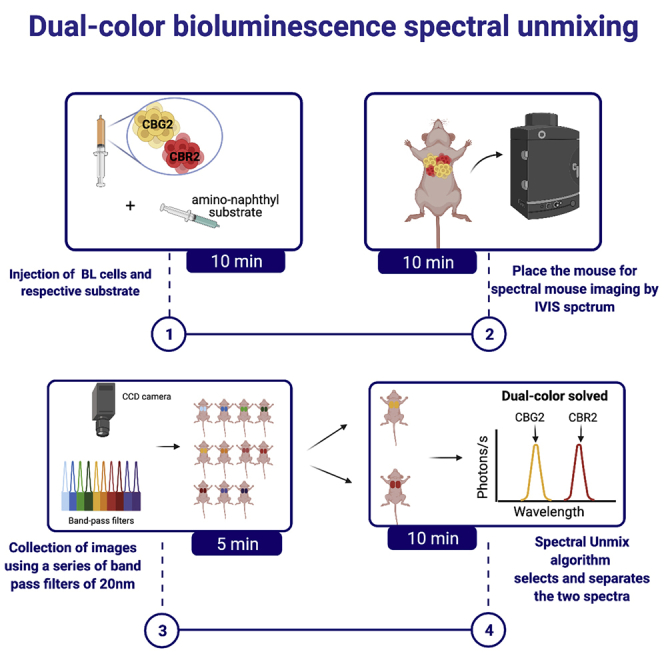

## Before you begin

### Experimental design considerations

Luciferase reporter genes provide a well-studied application for bioluminescence imaging of various biological molecular events both *in vitro* and *in vivo*. The production of bioluminescence (BL) light relies on the luciferase enzyme that catalyzes the oxidation of a luciferin substrate. Besides the traditional Fluc firefly luciferase reporter gene, new mutant red-shifted luciferases derived from fireflies or click beetles have been designed and developed recently (Zambito et al., 2021). Indeed, the signal attenuation due to absorption and scattering by tissues is reduced when red or near-infra-red luciferases are employed. Thus, the use of luciferases emitting in the so called “bio-optical window” (λ= 650 nm–800 nm) is highly desirable to improve imaging sensitivity (Mezzanotte et al., 2013 and Aswendt et al., 2019).

For multicolor BLI *in vivo*, the selection of luciferase pairs becomes crucial. Moreover, the administration of multiple substrates will require longer imaging sessions and challenging interpretation of data. The application of a single substrate that has affinity with multiple luciferases might be ideal (Stowe et al., 2019). The use of a cooled charged-coupled device (CCD) camera to acquire light emission and an adequate software to quantify light outputs is necessary to perform multicolor near-infrared BLI as described in this protocol.

**Figure 1:**
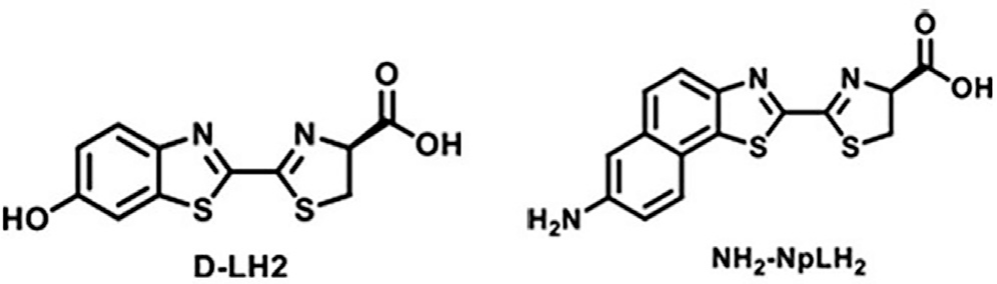
Chemical structures of D-LH_2_ luciferin and its analog NH_2_-NpLH2

Herein, we generated HEK-293T cells expressing equimolar amount of either CBG2 or CBR2 luciferase following the standard protocol for lentiviral transduction described in Knol-Blankevoort et al., 2016. HEK-CBG2 and HEK-CBR2 cells were tested with NH_2_-NpLH2 substrate as D-luciferin analog (Figure 1) (Hall et al., 2018). *In vitro* evaluations were performed to confirm efficiency of transduction and to calculate the expression rate of luciferase reporter genes (Zambito et al., 2021). For *in vivo* evaluations, HEK-CBG2 and HEK-CBR2 were injected intravenously and NH_2_-NpLH2 luciferin was injected intraperitoneally.

Colocalized bioluminescent light outputs from HEK-CBG2 and HEK-CBR2 are detected in the lungs. Separation of the two different spectra of emission and quantification of photon fluxes were performed by spectral unmixing algorithm tool, part of the Living Image software of the IVIS imaging Spectrum system (Perkin Elmer).

## Key resources table

**Table:** 

REAGENT or RESOURCE	SOURCE	IDENTIFIER
**Bacterial and virus strains**		

Lentivirus carrying EF1-CBR2opt-T2A-copGFP	Addgene	Plasmid #108713
Lentivirus carrying EF1-CBG2-T2A-copGFP	Mezzanotte Lab	N/A

**Chemicals, peptides, and recombinant proteins**		

NH_2_-NpLH2 substrate	Promega/synthesis reported in (Hall et al., 2018)	N/A
Isoflurane Isoflutek 1000 mg/g	Laboratorios Karizoo	710004
Dulbecco's Phosphate Buffered Saline (1×), (DPBS)	Lonza Bioscience	BE17-513F
Dulbecco’s modified Eagle’s medium (DMEM)	Gibco	11880028

**Experimental models: Cell lines**		

293T (HEK) cell line	ATCC	ATCC® CRL-3216™

**Experimental models: Organisms/strains**		

Nude mouse: BALB/C wild type	Charles River Laboratories (The Netherlands)	CAnN.Cg-*Foxn1*^*nu*^/Crl

**Software and algorithms**		

Living Image software 4.5 and above	PerkinElmer	Part #128113
GraphPad Prism 7 software	GraphPad	N/A

**Other**		

IVIS Spectrum Imager	PerkinElmer	124262
BC Insuline 1 mL U-100 29G, 0.33mm × 12.7mm 10× 10st, syringe, single use	BD Biosciences	Cat# 309623
Corning® 96 Well-Black Polystyrene Microplate	Greiner Bio-one	CLS3601-100EA
T75 EasYFlask, TC Surface, Solid Cap, Pack of 5	Thermo Fisher Scientific	156472

## Materials and equipment

HEK-CBG2 and HEK-CBR2 have been cultured in complete DMEM medium, which can be stored at 4°C for up to 1 month.

**Table:** 

Reagents	Final concentration (%)	Amount
Dulbecco’s modified Eagle’s medium (DMEM), stored at 4°C	89%	445 mL
FBS	10%	50 mL
Penicillin/streptomycin	1%	5 mL
Total	100%	500 mL

## Step-by-step method details

### *In vitro* spectral unmixing of two bioluminescent cell populations

**Timing: 3 days**

1. Plate mixtures of HEK-CBG2 and HEK-CBR2 cells in the following ratios: 100:0, 75:25, 50:50 for HEK-CBG2:HEKCBR2 and vice versa. Plate them in a 96-well black plate with clear bottom at the density of 1 × 10^4^ cells / 100 μL per well and incubated at 37°C for 24 h. For statistical analysis, two triplicates should be considered.
2. The next day, prepare NH_2_-NpLH2 substrate at final concentration of 2 mM in PBS.

**CRITICAL:** Keep the NH_2_-NpLH2 luciferin solution protected from light and store it at −20°C. If the color changes, discard and make fresh luciferin solution.

3. Prior to imaging the plate, replace DMEM culture media with PBS and resuspend the cells in 100 μL PBS per well. This will avoid interference by colored media while measuring the photon flux.
4. Add 10 μL NH_2_-NpLH2 substrate (2 mM) to each well containing plated cells in 200 μL PBS. Final concentration of NH_2_-NpLH2 substrate will be 0.1 mM.
  a. Start imaging 10 min after substrate addition to collect the maximum number of photons.
5. Set up imaging settings at the IVIS spectrum system with open filter, 30 s exposure time, field of view (FOV) C, f/stop=1, medium binning.
6. Select bandpass filters ranged from 580 nm to 800 nm to measure the spectra of interest (starting from the lowest to the highest filter)
  a. With the guided spectral library tool, create specific libraries for pure HEK-CBG2 cells (100%) and HEK-CBR2 cells (100%).
  b. Use the relevant libraries of HEK-CBG2 cells (100%) and HEK-CBR2 cells (100%) and apply them to perform the spectral unmixing in wells where unknown HEK-CBG2: HEK-CBR2 cell mixtures are plated.
  c. The resolved HEK-CBG2 and HEK-CBR2 emission will appear as separated images where quantification is possible.

***Note:*** A detailed guide on how to perform spectral unmixing is reported as technical note on Perkin Elmer website (https://resources.perkinelmer.com/labsolutions/resources/docs/TCH_011047_01_Spectral_Unmixing.pdf )

Normalize the emission spectra so that the CBG2 and CBR2 emission peaks can be visualized.

7. For well plate image analysis, calculate light outputs for the HEK-CBG2 and HEK-CBR2 emission by drawing Region Of Interest (ROI) by Living image software 4.5 (Perkin Elmer). Place a ROI over the signal in each well and select “measure”. The software will record the BL outputs in photon flux (photons/sec).
8. Export data outputs to Excel (Microsoft) sheet to measure the average amongst triplicates and relative standard deviations.

***Note:*** the substrate is light sensitive and must be kept in the dark. We recommend receiving training on using the IVIS Spectrum.

### *In vivo* spectral unmixing of two cell populations

**Timing: 2 days**

9. Culture separately HEK-CBG2 and HEK-CBR2 cells in a T75 flask (cell density ∼ 1× 10^6^/mL) and incubate at 37°C for 24 h.
10. The next day, prepare NH_2_-NpLH2 substrate solubilized in PBS at the final concentration of 44 mg mL^−1^ (4.4 mg NH_2_-NpLH2 in 100 μL of PBS per 20 g mouse corresponds to a dose of 220 mg Kg^−1^).

**CRITICAL:** Keep NH2-NpLH2 luciferin solution protected from light and. If the color changes, discard and make fresh luciferin solution.

**Figure 2:**
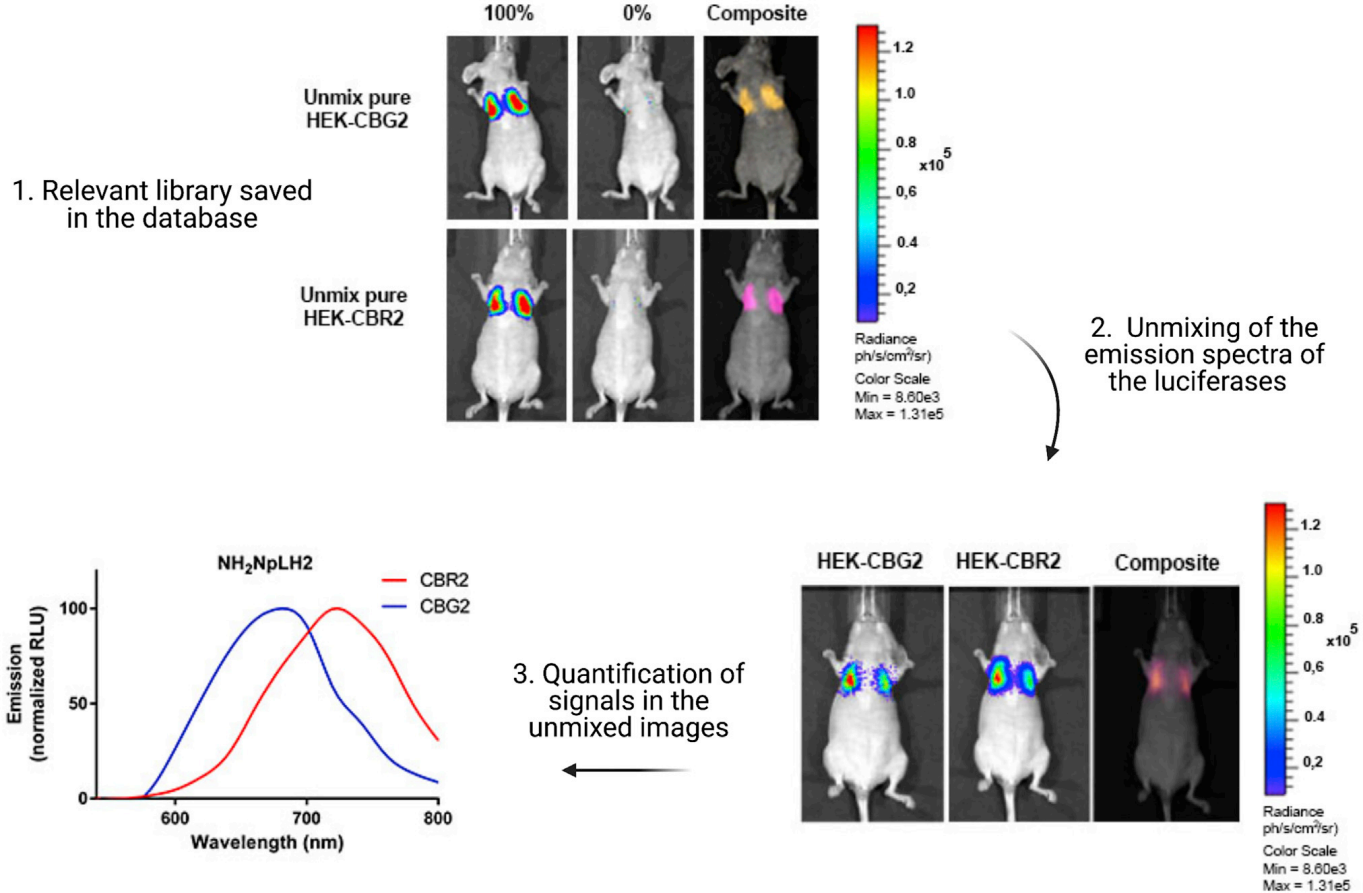
Illustration of the spectral unmixing for co-localized bioluminescent signals *in vivo* 1) Create and save the specific libraries for pure (100% cell ratio) HEK-CBG2 and HEK-CBR2 in the database. 2) Use the relevant libraries to unmix the emission outputs when HEK-CBG2 and HEK-CBR2 are co-injected i.v. in the same mouse. 3) Use the spectral unmixing algorithm to separate and quantify the unmixed images. Normalized emission curves can be plotted highlighting the luciferase emission peaks. Scale bar: Radiance (ph/s/cm^2^/sr).

**Figure 3:**
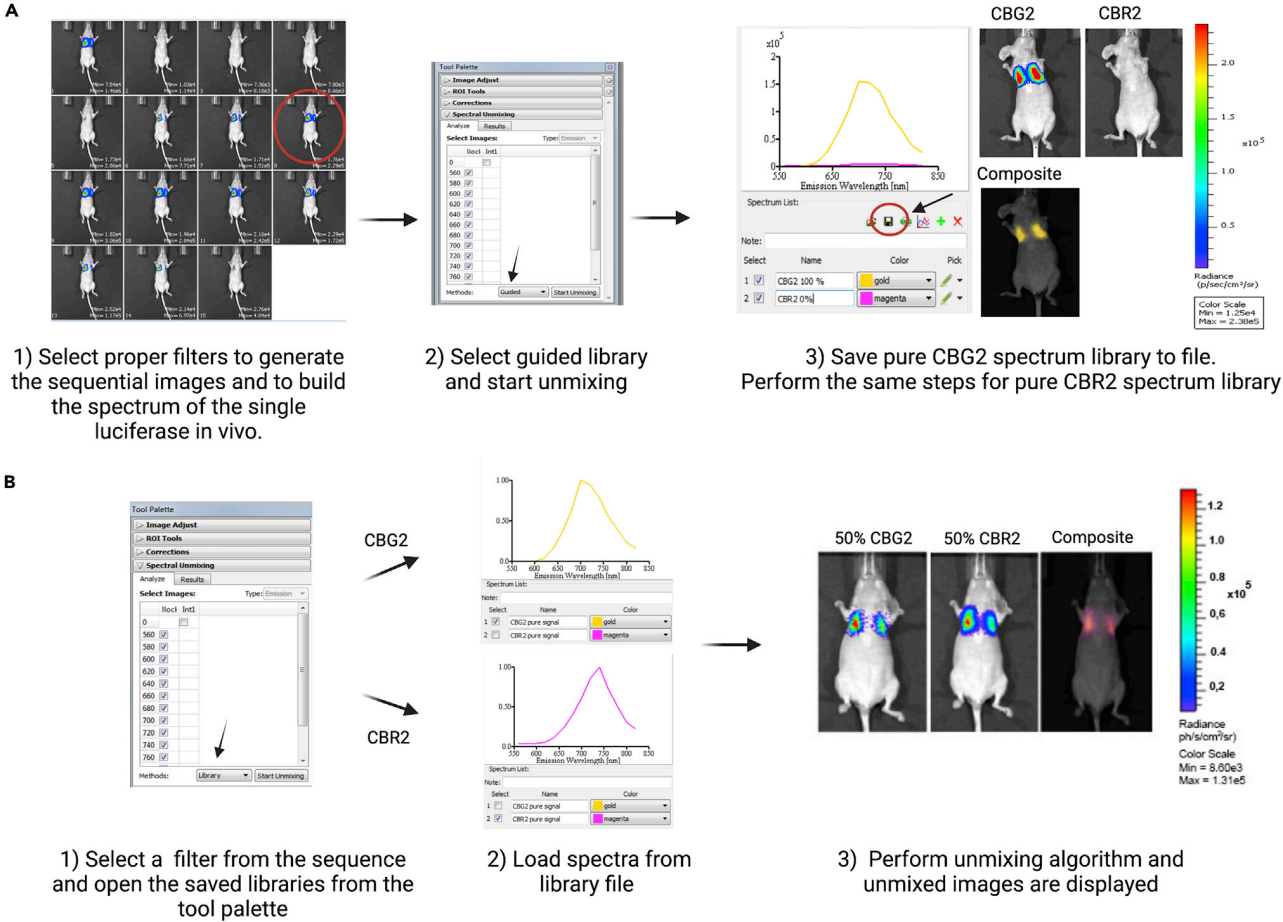
Step-by-step spectral unmixing (A) Save the specific luciferase spectrum to create a pure (100% cell ratio) HEK-CBG2 or HEK-CBR2 library. (B) Perform spectral unmixing by loading opportune pure libraries. The algorithm will be able to distinguish each luciferase contribution even if the two luciferases are colocalized in the same area. Scale bar: radiance (ph/s/cm^2^/sr). A detailed guide on spectral unmixing is reported as technical note on Perkin Elmer website.

11. Prior *in vivo* imaging, wash HEK-CBG2 and HEK-CBR2 cells twice with DPBS and resuspend them in sterile DPBS.
  a. Prepare cell aliquots (final concentration 1 × 10^6^ /100 μL sterile DPBS per mouse) for 100% HEK-CBG2, 100% HEK-CBR2 and for various ratios for HEK-CBG2: HEK-CBR2 and vice versa.
12. Set up imaging settings at the IVIS spectrum imager system with open filter, 30 s exposure time, field of view (FOV) C, f/stop=1, medium binning.
  a. Select bandpass filters ranged from 540 nm to 800 nm to measure the spectra of interest (starting from the lowest to the highest filter)

13. Register guided spectral libraries of 100% luciferase-HEK293T (Figure 2):
  a. Anesthetize BALB/C nude mice (females, aged 6–10 weeks) with 1.5%–2.0% isoflurane (flow rate 1 L/min O_2_).
  b. Inject 100 μL 100% HEK-CBG2 or 100% HEK-CBR2 cells with intravenous injections (I.V.)
  c. Inject 220 mg Kg^−1^ of NH_2_-NpLH2 substrate solubilized in DPBS intraperitoneally (I.P.)
    ***Note:*** We recommend pre-loading one syringe per mouse. All mice must be injected with luciferin within a few seconds of each other. This also reduces differences in dosage and enhance the accuracy.
  d. Perform the spectral imaging 10 min post I.P. injection to measure the highest light output.
  e. With the guided spectral library tool (in the ROI menu), create specific libraries for pure *in vivo* signals for HEK-CBG2 injected (100%) and for HEK-CBR2 injected (100%) (Figure 3).

14. Spectral unmixing for unknown HEK-CBG2: HEK-CBR2 cell ratios:
  a. Anesthetize nude mice with 1.5%–2.0% isoflurane (flow rate 1 L/min O_2_).
  b. Inject 100 μL HEK-CBG2: HEK-CBR2 and vice versa at different cell ratios with intravenous injections (I.V.)
  c. Perform the spectral imaging 10 min post i.p. injection to let the substrate stabilize and allow the measurement of highest light outputs.
  d. After imaging, the mice can be placed back into their respective cages. They should be awake within 30 s to 1 min.
  e. For spectral imaging, choose band pass filters between 620–800 nm and select the relevant guided library registered for *in vivo* spectra of HEK-CBG2 cells (100%) or HEK-CBR2 cells (100%) on the ROI menu.
  f. Apply the relevant libraries to perform the spectral unmixing for unknown HEK-CBG2: HEK-CBR2 cell ratios and draw a mask where the cell mixtures are expected to be localized after i.v. injections
  g. Proceed with spectral unmixing, the algorithm will separate HEK-CBG2 and HEK-CBR2 cell contributions
  h. Two unmixed images will be built each containing the signature of only one of the luciferases of interest.
15. To calculate light outputs for the unmixed images, draw Region of Interest (ROI) by Living image software 4.5 (Perkin Elmer). Place a ROI over the signal.
16. Export data outputs to Excel (Microsoft) to measure the average amongst triplicates and relative standard deviations. Graph the “total flux” column.
17. Perform the *in vivo* experiment with at least three mice

**CRITICAL:** Ear tags are necessary to distinguish mice during the luciferin injections.

***Note:*** Up to five mice can be imaged and keep the stage heated to 37°C during the *in vivo* imaging. Total imaging time acquisition is about 6 min.

We recommend receiving training on using the IVIS Spectrum as well as its accompanying isoflurane anesthesia machine.

## Expected outcomes

Simultaneous monitoring of bioluminescent cell populations in the same animal model confers the great advantage of visualizing cell behavior in their environment. Previously, we have attempted this method by developing CBG2 luciferase that could be used for dual-color BLI *in vivo* and in the lungs as deep tissue model. CBG2 can be paired with CBR2 and both have affinity for NH_2_-NpLH2 substrate. HEK-293T cells expressing CBR2 or CBG2 luciferases can be injected at various percentages *in vivo*. We recommend to use nude mice to enhance the sensitivity of measurement of photon flux *in vivo*.

Spectral unmixing algorithm enables sensitive quantification of bioluminescent signals. We utilized this method to specifically discriminate CBR2 and CBG2 bioluminescent emissions when co-localized in the same area which is the most challenging application. The two BL spectra can be accurately separated and the photon fluxes can be measured for each selected band-pass filter. This technique allows to reduce imaging time sessions and to refine the number of animal models required for the experimental test.

This technique could also be extended to perform *in vivo* multi-color BLI where another luciferase/luciferin system can be added to our proposed protocol for CBG2 and CBR2 with NH_2_-NpLH2 substrate. We advise to select an orthogonal system like Nanoluc marine luciferase that has specific affinity for coelenterazine-like substrates but not for D-luciferin-like substrates. This will ensure an enhanced spectral separation for each luciferase used.

However, when the same animal is imaged with two different D-luciferin analogs, selected luciferase enzymes must have low detectable light outputs for one of the substrates employed. This will guarantee a successful spectral unmixing. Indeed, substrates must preserve the color modulation and must prevent color-shift greater than 40 nm *in vivo*. Imaging sessions with two different D-luciferin analogs must be performed at least after 4 h from the first imaging session to allow full clearance of the substrate.

Although we have attempted the spectral unmixing for lung imaging model, we expect the method to be accurate for dual-color imaging of smaller areas (e.g., lymph nodes and cell depots cells in deep organs like brain).

## Quantification and statistical analysis

*In vitro* and *in vivo* tests were performed using GraphPad 7 software for ONE-way ANOVA followed by a post-test for column analysis. Results reported as mean ± SD and significance attributed when p< 0.001 (∗) for *in vitro* experiments or p< 0.05 (∗) for *in vivo* experiments.

## Limitations

It has been noted that a signal limit for spectral unmixing does not exist. However, for accurate quantification we recommend more than 600 counts for all signals in the area to unmix. If the signals are less than 600 counts, background noise will affect the reliability of the quantification. To delineate precise luciferase spectral curve and relative peak of emission, we advise to register also marginal band-pass filters.

## Troubleshooting

### Problem 1

The spectral library built *in vitro* has a different spectral peak compared to the spectral library built *in vivo* (in vivo spectral unmixing of two cell populations, step 14).

### Potential solution

Spectral emission *in vitro* can differ from the spectral emission *in vivo*. This is because tissue components and deep-tissue imaging can alter the detection of photons registered for each band-pass filter. Indeed, we observed a partial shift of the spectral peak for CBG2 luciferase (from 650 to ∼ 680–700 nm) due to the partially absorbed green photons when imaging in depth. Please build your spectral library for *in vitro* and *in vivo* separately.

### Problem 2

Spectral library can be built *in vitro* but not *in vivo* (in vivo spectral unmixing of two cell populations, step 13e)

### Potential solution

Luciferases selected for the dual-color system must have comparable photon yield *in vivo*. It is essential to register first the guided library of pure luciferases *in vivo*. In general, bright and red-shifted luciferases are the best choice for deep tissue imaging. If the spectral emissions are completely overlapping, the spectral unmixing tool will not be able to discriminate each luciferase signature.

### Problem 3

Signal background occurs *in vivo* after NH_2_-NpLH2 substrate i.p. injection (in vivo spectral unmixing of two cell populations, step 13 d).

### Potential solution

We observed low background derived from NH_2_-NpLH2 substrate after i.p.injection in the liver. If this is the case and you need to unmix signal in the liver quantify the background first. If your signals are 10 or 100 times higher than background emission then it can be considered negligible and it will not affect your quantification after unmixing.

### Problem 4

Multiple *in vivo* administration of NH_2_-NpLH2 substrate (in vivo spectral unmixing of two cell populations, step 13 c).

### Potential solution

NH_2_-NpLH2 substrate is not toxic and multiple injections in the same day are possible. Prior imaging, mice should be first checked for BL background and for complete substrate clearance. However, we do not recommend repeated isoflurane anesthesia in a short frame time because this might be toxic and mouse discomfort should be prevented.

### Problem 5

Mice preparation for *in vivo* imaging (in vivo spectral unmixing of two cell populations, step 13 a)

### Potential solution

Animal hair can highly affect optical imaging by blocking, absorbing, and scattering the light. The near-infrared spectrum reduces this phenomenon but typically shows minimal scattering and absorbance of light. We recommend removing the fur around the area that needs to be imaged and continuing this practice throughout the study.

Nude mice have the advantage that does not require depilation and they are preferred for optical imaging techniques.

## Resource availability

### Lead contact

Further information and requests for resources and reagents should be directed to and will be fulfilled by the lead contact, Laura Mezzanotte, Dr. l.mezzanotte@erasmusmc.nl

### Materials availability

Lentiviral construct for generation of lentivirus carrying EF1-CBG2-T2A-copGFP or EF1-CBR2-T2A-copGFP are available from the lab upon request. NH_2_-NpLH2 substrate is available on request from Promega Corporation.

### Data and code availability

The protocol does not include all datasets generated or analyzed during this study.
